# Predicting Amputation in Patients With Diabetic Foot Ulcers: A Systematic Review

**DOI:** 10.7759/cureus.27245

**Published:** 2022-07-25

**Authors:** Zahraa Mansoor, Ali Modaweb

**Affiliations:** 1 Pediatrics, Al Jalila Children’s Specialty Hospital, Dubai, ARE

**Keywords:** peripheral arterial diseases, diabetic foot ulceration, foot ulcers, lower limb amputation, type-2 diabetes mellitus

## Abstract

Foot ulcers are a leading cause of morbidity in diabetics. One of the known complications of diabetic foot ulcers is lower limb amputation which makes it a major socioeconomic problem. Currently, there’s a lack of knowledge on the predictors of amputations in diabetics with foot ulcers. We performed a systematic review of studies that identified risk factors of amputation in patients with diabetic foot ulcers. This systematic review aims to identify the predictors of amputation in order to optimize the management strategy and care plan. Medline database was searched and inclusion criteria were implemented for the selection of studies. The risk factors extracted were part of four categories: (i) history and physical examination, (ii) ulcer characteristics, (iii) lab results, and (iv) co-morbidities. The data extracted were in the form of odds ratios, 95% confidence intervals, and predictive values. The mean values with standard deviations of the included risk factors were recorded, and the incidence of risk factors among the amputation groups was identified or calculated when the data were sufficient. Seven articles were selected reporting on 3481 patients. This review identified peripheral arterial disease, neuropathy, high Wagner’s grade, osteomyelitis, postprandial glucose level, white cell count, c-reactive protein, erythrocyte sedimentation rate, low hemoglobin, and albumin as the most significant predictors of amputation.

## Introduction and background

Diabetes mellitus is one of the most prevalent metabolic disorders that cause significant morbidity among the population. The global burden of diabetes can be estimated by its mortality rate of which most is attributed to the complications it causes; including cardiovascular, renal, and gestational problems. According to the International Diabetes Federation, 4.6 million diabetes-related deaths were recorded in 2011, accounting for 8.2% of all-cause mortality [1]. Among the leading causes of morbidity in diabetics are foot ulcers, usually preceded by peripheral arterial disease, neuropathy and trauma, or by a combination of all three along with other factors. Nearly a quarter of the population of diabetics develop a foot ulcer at some point in their life [2], making it a serious inevitable health and socioeconomic problem considering that 12% of those require an amputation [3]. Diabetics with foot ulcers have an eight-fold higher risk of a lower extremity amputation compared to non-diabetics [4], and about 50% of diabetics who undergo amputations die within five years [5]. This adds a heavy burden on both individual and social scales; it increases disability, reduces the quality of life, generates undesirable psychological impacts, increases medical costs, and creates a massive economical load on the healthcare system [6]. Therefore, the indicators of progression of an ulcer to a mandatory amputation must be discovered at an early stage, and several aspects should be considered for saving the limb before attempting amputation. The main focus of this systematic review is to identify the factors that can predict an increased risk of amputations and to determine the most common/strongest predictor among all in order to ensure early identification of risk. It is highly crucial for these predictors to be recognized as many patients undergo unnecessary amputations due to lack of diagnostic efficiency and inadequate clinical judgment, thus needlessly endure an unfortunate experience [7]. These predictors are essential to optimize the management strategy and care plan for these patients, and they can be very helpful in preserving the limb and avoiding disastrous circumstances. Few systematic reviews have been conducted to tackle similar issues, and most of them focus on the risk of amputations in terms of one variable only, e.g., the effect of peripheral arterial disease [8]. Our systematic review reports different variables as the risk factors vary inconsiderably among patients. Furthermore, there’s a lack of a standardized predictive model used among healthcare professionals to predict the risk of amputation, and the current guidelines for prevention do not incorporate the predictors found in different studies and articles [9]. This systematic review aims to predict the strongest factors that can anticipate the risk of amputation in diabetic foot ulcers, for it to be treated and reversed before permanent damage occurs.

## Review

Methods

Search Strategy and Study Selection

**Figure 1:**
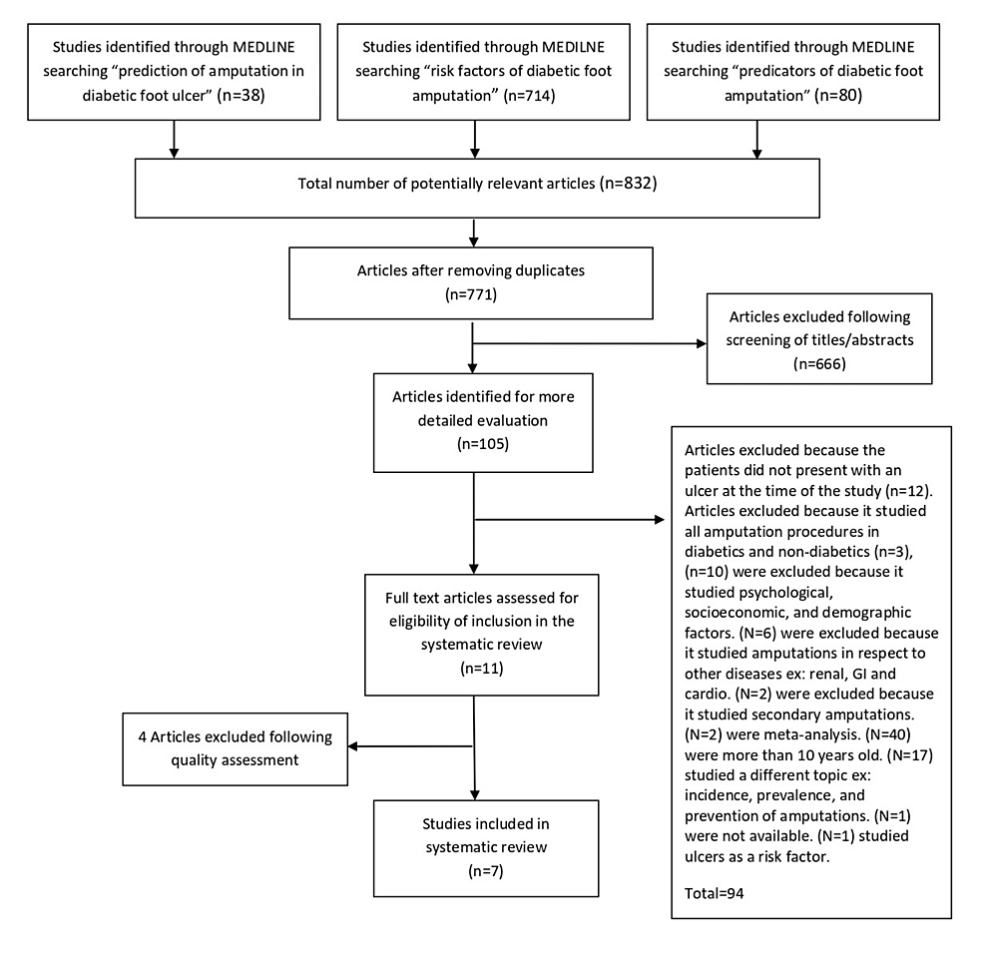
Systematic review PRISMA flow chart for study selection process. PRISMA: Preferred Reporting Items for Systematic Reviews and Meta-Analyses

This systematic review was performed based on the guidelines in the Preferred Reporting Items for Systematic Reviews and Meta-Analyses (PRISMA) checklist [10]. An electronic search strategy was implemented; MEDLINE database was searched for English articles reporting risk factors of amputations in diabetic foot ulcers published in the past 10 years. First, three searches were conducted in MEDLINE using different search phrases, then the results of all three were combined (Figure 1). After the removal of duplicates, the remaining articles were assessed by screening titles to determine the potentially relevant studies. The abstracts of the identified studies were evaluated for eligibility through the inclusion criteria by two reviewers independently. At last, the final studies that met the inclusion criteria were assessed for quality using Newcastle-Ottawa scale (NOS) for non-randomized controlled trials (Tables 1, 2). Articles that received less than 5/9 in the quality assessment were excluded; all the other articles were included in the systematic review.

**Table 1:** Quality assessment of cohort and cross-sectional studies.

Study	Score										Included/excluded
	Selection				Comparability		Outcome			Total no. of points	
	Representation of exposed patients (no. of points)	Selection of unexposed patients (no. of points)	Ascertainment of exposure (no. points)	Outcome was not present at start (no. of points)	Symptoms are controlled (no. of points)	Therapy of patients controlled (no. of points)	Assessment of outcome (no. points)	Follow-up long enough for outcome to occur (no. of points)	Adequacy of follow-up of cohorts (no. of points)		
Yesil et al. 2009 [11]	1	0	0	1	1	1	1	0	1	7/9	Included
Sun et al. 2012 [12]	1	1	1	0	1	1	1	0	0	6/9	Included
Namgoong et al. 2016 [13]	1	1	1	1	1	1	1	0	0	7/9	Included
Jiang et al. 2015 [14]	1	1	1	1	1	0	1	0	0	6/9	Included
Zubair et al. 2012 [15]	1	0	1	1	1	1	1	0	0	6/9	Included
Li et al. 2011 [16]	1	1	1	0	1	1	1	0	0	6/9	Included

**Table 2:** Quality assessment of case-control studies.

Study	Score									Included/excluded
	Selection				Comparability	Exposure			Total number of points	
	Is the case definition adequate?	Representativeness of the cases	Selection of controls	Definition of controls	Comparability of cases and controls on the basis of the design or analysis	Ascertainment of exposure	Same method of ascertainment for cases and controls	Non-response rate		
Pemayun et al. 2015 [17]	1	1	1	0	1	1	1	0	6/8	Included

Criteria for Including Studies

The studies were included based on the following criteria: (1) published observational studies (cohort, case-control, and cross-sectional); (2) English articles; (3) published in the past 10 years; (4) type 1 or 2 diabetic population; (5) all patients must present with an ulcer at the time of the study; (6) primary outcome: amputation; (7) all studies tested risk factors for amputation; and (8) sample size: any.

Data Extraction

Certain data were selected from every section of the included studies. From the front page/abstract - the title, date, and the country of the study were identified. From the methods section, the study type, sample size, the measured variables, and the methods of measurements were collected. The risk factors extracted from the results section were part of the following four categories: (i) history and physical examination, (ii) ulcer characteristics, (iii) lab results, and (iv) co-morbidities. All of the data extracted were in the form of odds ratios, 95% confidence intervals, and predictive values. The mean values with standard deviations of the included risk factors were recorded when available, and the incidence of risk factors among the amputation groups was identified or calculated when the data were sufficient. The data were either presented in graphs or extracted following a detailed evaluation of the text. The number of amputations, overall amputation rate, mean age, and predominant gender of the amputation group were documented in every study.

Data Analysis

The data were presented in the form of odds ratios with p-values and confidence intervals. Risk factors with p-values less than 0.05 were considered statistically significant. A forest plot of the most common predictors was performed. Data were analyzed using the application Comprehensive Meta-Analysis version 3 (Frederick, MD: Biostat, Inc.).

Results

Search Results

The study selection strategy was applied on 832 articles as shown in Figure 1. A total of 11 articles met the inclusion criteria and were further evaluated by the Newcastle-Ottawa score. Finally, seven articles were selected for the systematic review by two reviewers. Disagreements between reviewers were resolved by discussion. Quality assessment of the non-randomized controlled trials was done using Newcastle-Ottawa scale (NOS).

Study Characteristics

**Figure 2:**
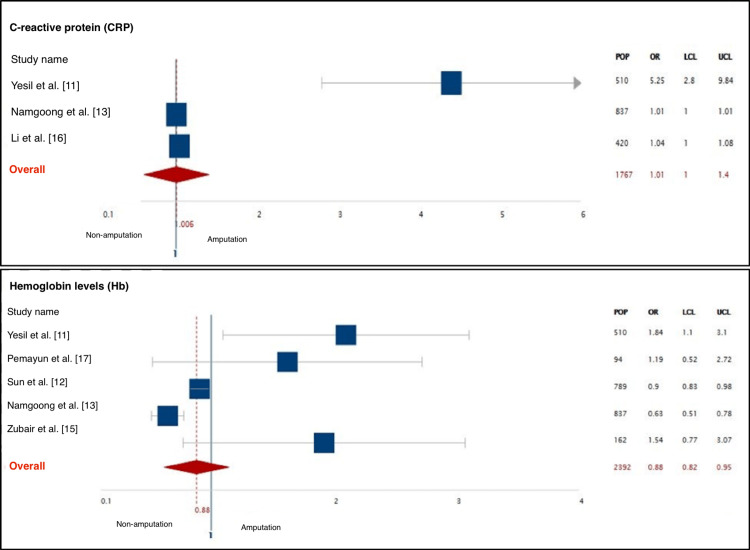
A meta-analysis of the most significant predictors of amputation. POP: population; LCL: lower confidence limit; UCL: upper confidence limit

**Figure 3:**
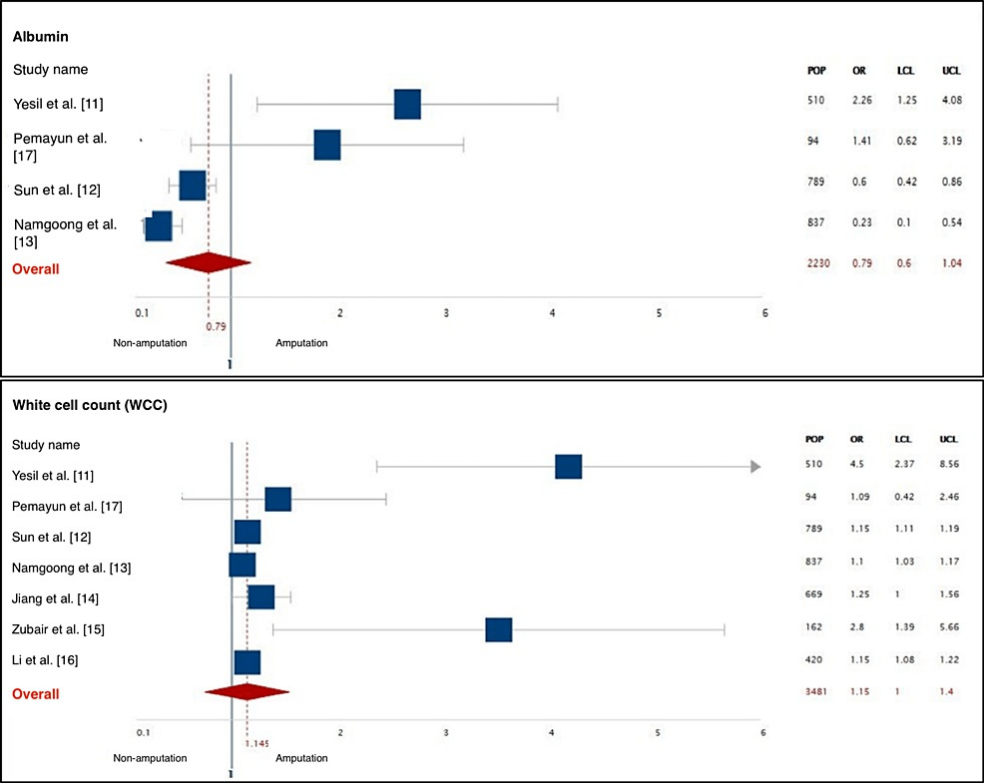
A meta-analysis of the most significant predictors of amputation showing albumin and WCC. POP: population; LCL: lower confidence limit; UCL: upper confidence limit; WCC: white cell count

**Figure 4:**
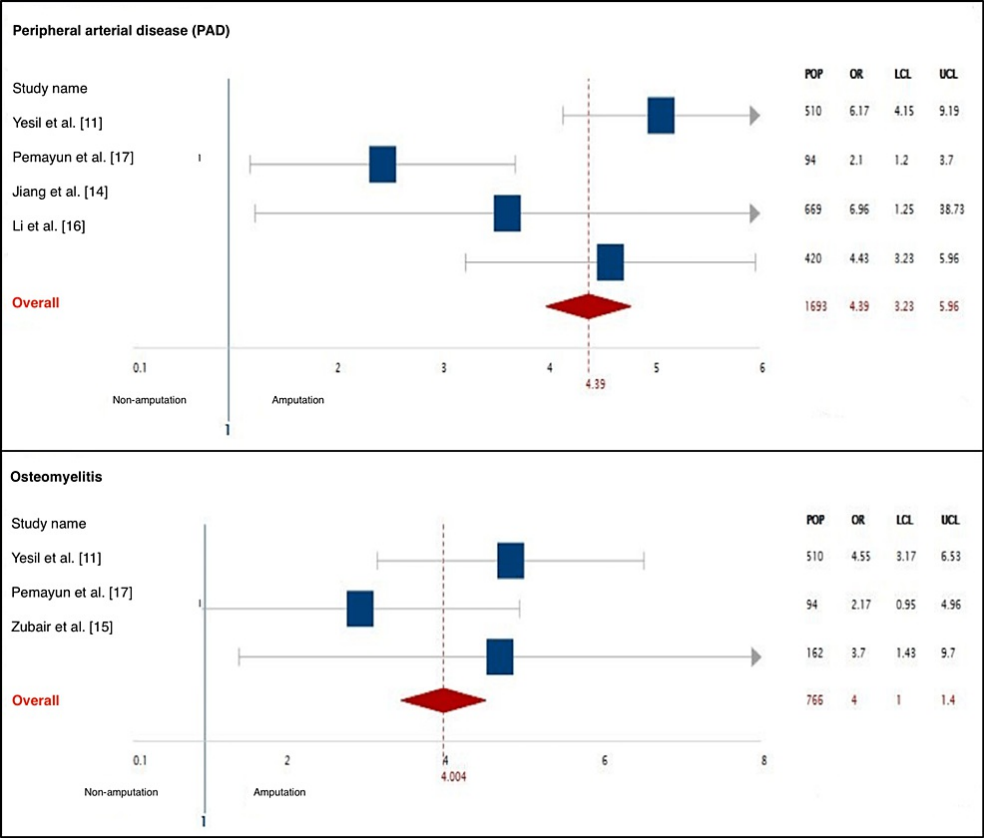
A meta-analysis of the most significant predictors of amputation showing PAD and osteomyelitis. POP: population; LCL: lower confidence limit; UCL: upper confidence limit; PAD: peripheral arterial disease

This systematic review consists of seven articles reporting on 3481 patients. Four studies were cohort, one study was case-control and two studies were cross-sectional. All the studied population involved patients with type 1 or 2 diabetes presenting with a foot ulcer, and the sample size ranged from 94 in small studies to 837 in large studies. The mean population size was 497.2. A total of 917 patients were identified as the amputation group, the indications of amputation were classified into four categories and retrieved from this group. The mean age of the amputation group was 60 and above, with males being the predominant gender. Further data regarding the incidence of amputation and overall amputation rate were either documented or calculated from every study. The overall amputation rate ranged from 3.34% to 42.83% (Table 3). In addition, summary of the most significant characteristics of patients undergoing amputations in the included studies, along with their odds ratios, 95% confidence intervals, and predictive values are presented in Table 4. Also, a meta-analysis was carried out on the most significant factors (Figures 2-4).

**Table 3:** Summarizing the characteristics of the included studies and comparing the overall amputation rates.

Study author	Yesil et al. 2009 [11]	Sun et al. 2012 [12]	Namgoong et al. 2016 [13]	Jiang et al. 2015 [14]	Zubair et al. 2012 [15]	Li et al. 2011 [16]	Pemayun et al. 2015 [17]
Country of study	Turkey	Taiwan	Korea	China	India	China	Indonesia
Type of study	Prospective cohort	Cross-sectional	Prospective cohort	Cohort	Prospective cohort	Cross-sectional	Case-control
Population size	510	789	837	669	162	420	94
Number of amputations	213	338	28	133	46	112	47
Overall amputation rate	37.11%	42.83%	3.34%	19.88%	28.4%	21.54%	Not mentioned

**Table 4:** Summary of the most significant characteristics of patients undergoing amputations in the included studies. FBG: fasting blood glucose; HbA1c: hemoglobin A1c; CRP: c-reactive protein; ESR: erythrocyte sedimentation rate; ABI: ankle-brachial index; PAD: peripheral arterial disease; FPG: fasting plasma glucose

Study	No. of amputations	Predominant gender	Average age of amputation group	Most significant predictors of lower extremity amputation found in the study					
				Factor	Mean	Incidence	OR	95% CI	p-Value
Yesil et al. 2009 [11]	213	72.3% male (154)	64.60±9.69 years	PAD	-	80.8%	6.174	4.149-9.188	<0.001
				Osteomyelitis	-	62.4%	4.55	3.172-6.526	<0.001
				High WBC (10^3^/mcL)	12.56±5.55x10^3^/mcL	-	4.504	2.371-8.556	<0.001
				ESR (mm/h)	73.87±32.41 mm/h	-	3.871	2.208-6.787	<0.001
				CRP (mg/dL)	108.76±90.22 mg/dL	-	5.25	2.801-9.842	<0.001
				High Wagner grade	-	58.3%	23.959	14.043-40.878	<0.001
				Neuropathy	-	77.5%	0.466	0.296-0.732	0.001
				Low Hb (g/dL)	11.13±19 g/dL	-	1.843	1.095-3.102	0.021
				Low albumin (g/dL)	3.55±0.54 g/dL	-	2.255	1.247-4.067	0.007
				Smoking	-	45.5%	1.412	1.003-1.986	0.048
Sun et al. 2012 [12]	338	56.2% male (190)	66.55 years	Low ABI	0.86	-	0.42	0.27-0.67	0.0002
				Low serum albumin	2.985 g/dL	-	0.60	0.42-0.86	0.0046
				Low Hb	9.45 mg/dL	-	0.90	0.83-0.98	<0.01
				High WBC (10^3^/mcL)	13750x10^3^/mcL	-	1.15	1.11-1.19	<0.0001
				High-grade Wagner classification	-	88%	13.10	8.74-19.65	<0.0001
				Neuropathy	-	84.02%	-	-	0.004
Namgoong et al. 2016 [13]	28	Male	-	Dialysis	-	-	8.683	2.834-26.601	<0.001
				GI disorders	-	-	6.740	1.175-38.66	0.032
				Ulcer invasion to bone	-	-	11.673	1.425-95.619	0.022
				Ulcer on hind foot area	-	-	6.158	1.808-20.974	0.004
				Low Hb	-	-	0.641	0.472-0.871	0.005
				High FBG	-	-	1.007	1.001-1.013	0.030
				Neuropathy	-	-	0.394	0.170.06-0.882	0.023
				Nephropathy	-	-	2.536	1.189-5.408	0.016
				High WBCs	-	-	1.098	1.034=1.167	0.002
				ESR	-	-	1.014	1.001-1.027	0.038
				CRP	-	-	1.006	1.003-1.01	<0.001
				Low albumin	-	-	0.23	0.098-0.541	0.001
				High creatinine	-	-	1.188	1.078-1.308	0.001
				Postprandial blood glucose	-	-	1.005	1-1.01	0.034
Jiang et al. 2015 [14]	133	Male	62.25 years	Foot deformity	-	20.3%	1.973	1.025-3.800	0.042
				Ulcer history	-	39%	1.973	1.009-46.209	0.049
				Revascularization history	-	14.2%	2.662	1.115-6.352	0.027
				Infection	-	84.9%	2.323	1.028-5.251	0.043
				Low postprandial blood glucose	13.5	-	0.941	0.885-0.999	0.048
				Increased duration of diabetes	126.5 months	-	1.004	1.000-1.007	0.026
				High WBC	9.85x10^9^	-	1.250	1.002-1.559	0.048
				Low albumin	11.5 mg/dL	-	-	-	0.006
				Low Hb	11.5 mg/dL	-	-	-	0.006
				Smoking	-	46.6%	-	-	0.018
Zubair et al. 2012 [15]	46	80.4% male (37)	49.8±13.6 years	Hypertension	-	73.9%	2.83	1.33-6.01	0.009
				Neuropathy	-	69.5%	3.01	1.45-6.24	0.002
				Nephropathy	-	58.6%	2.24	1.18-4.49	0.02
				PAD	-	10.6%	6.95	1.2-37.2	0.02
				High ulcer grade≥2 (Texas grade)	-	63%	3.7	1.43-9.7	0.007
				High WBC (10^3^/mcL)	9.59±3.4x10^3^/mcL	58.6%	2.80	1.39-5.66	<0.004
				High cholesterol (>150 mg/dL)	183.6±36.4 mg/dL	54.3%	3.74	1.82-7.68	0.0003
				High triglycerides (>200 mg/dL)	164.6±102.5 mg/dL	58.6%	5.44	2.6-11.4	<0.005
				Biofilm infection	-	84.7%	4.52	1.87-10.9	<0.0008
				Previous antibiotics use	-	76%	9.12	4.11-20.1	<0.001
				High Creatinine (>1.5 mg/dL)	1.45±0.67 mg/dL	50%	3.46	1.67-7.4	<0.0004
				Increased duration of diabetes	13.3±5.7 years	-	-	-	<0.001
Li et al. 2011 [16]	112	64.2% male (72)	65.7±10.7 years	High WBC (10^9^/L)	10.8±6.5x10^9^/L	99.1%	1.146	1.075-1.222	<0.001
				CRP (mg/L)	9.95±7.45 mg/L	91.9%	1.041	1.002-1.082	0.037
				PAD	-	93.7%	4.529	1.500-13.676	0.007
				Low triglycerides (mmol/L)	1.16±0.60 mmol/L	96.4%	0.614	0.433-0.869	0.006
				Low Hb (g/L)	10.78±18.4 g/L	100%	-	-	0.002
				High cholesterol (mmol/L)	4.21±0.92 mmol/L	96.4%	-	-	≤0.001
Pemayun et al. 2015 [17]	47	59.6% female (28)	52.6±7 years	Hypertension	-	65.9%	3.67	1.14-11.79	0.028
				Presence of PAD	-	61.7%	12.97	3.44-48.88	<0.001
				FPG≥126 mg/dL	-	97.8%	8.67	0.74-101.11	0.085
				Triglycerides≥150 mg/dL	-	70.2%	5.58	1.74-17.91	0.004
				HbA1c≥8%	11.3±2.8%	95.7%	20.47	3.12-134.31	0.002
				High Wagner grade (≥3)	-	95.7%	25.88	6.97-96.13	<0.001

History and Physical Examination

All of the included studies tested the influence of age, gender, smoking, and duration of diabetes on the outcome of foot ulcers. Most of the amputated patients were in the age group of 60 and above. According to the odds ratio and p-value, only two studies provided evidence that the risk of amputation increased with age, with the highest odds ratio being (OR: 1.732, 95% CI: 1.099-2.730) in the study of Yesil et al., in the age group of 64.6 (±9.69 SD) [11]. As to gender, the number of males among the amputation group exceeded the number of females as they were predominant in six out of seven studies. The prevalence of males in the amputation groups ranged from 8.4% to 64.2%, and the factor of gender was considered significant in the study of Zubair et al. only according to the p-values (0.006) [15]. Pemayun et al. was the only study that stated a higher number of females [17]. A statistical significance of both smoking and the duration of diabetes was displayed in two studies only, the prevalence of smoking was approximately 45.5% in the study of Yesil et al. and 46.6% in Jiang et al. while the duration of diabetes in the amputation group ranged from five to 17 years [11,14]. The duration of diabetes was considered significant in Sun et al. (p=0.03) and Zubair et al. (<0.001) [12,15]. On the other hand, the BMI of the patients was calculated in one study only by Yesil et al., and it appeared to be significantly lower in the amputation group (p=0.002) with mean BMI of 25.56±3.73 [11]. The remaining studies had no data regarding BMI.

Laboratory Results

**Figure 5:**
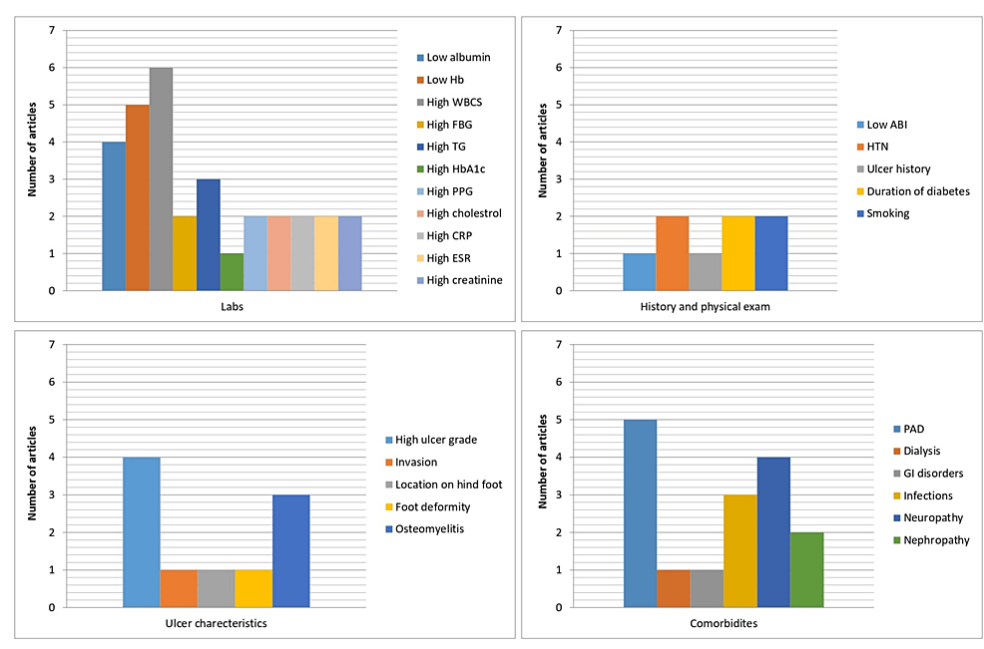
Demonstration of the significant factors in the included studies. The images show the number of studies (Y-axis) where risk factors (X-axis) were significant. FBG: fasting blood glucose; TG: triglycerides; HbA1c: hemoglobin A1c; PPG: postprandial glucose; CRP: c-reactive protein; ESR: erythrocyte sedimentation rate; ABI: ankle-brachial index; PAD: peripheral arterial disease; HTN: hypertension

The studies included in the review evaluated several serological and biochemical markers for the prediction of amputation. Both glucose and non-glucose-related markers were included to assess the outcome including hemoglobin A1c (HbA1c) which was only significant in the study of Pemayun et al., as 95.7% of the amputation group had a high HbA1c (mean value 11.3±2.8%) [17]. Both fasting plasma glucose and two-hour postprandial glucose (PPG) were significant in two studies only. On the other hand, The most significant variables identified in this systematic review out of the laboratory results category were as follows: (1) albumin, five out of seven articles demonstrated the efficacy of albumin in predicting amputations among diabetics. Most patients undergoing amputations had a lower level of albumin (<2.9 g/dL). (2) Hemoglobin (Hb), It was evident in 6/7 articles that the amputation group had lower levels of Hb, which correlates significantly to the strength of Hb measurement in predicting amputations (<13.5 g/dL). (3) White cell count (WCC), 6/7 studies displayed that the amputation group had high WCC with the highest odds ratio in the study of Yesil et al. with a mean WCC of 12.56±5.55x10000/mcL (OR: 4.504, p=0.001, 95% CI: 2.371-8.556). On the other hand, the predictive value of triglycerides, cholesterol, c-reactive protein (CRP), and creatinine was significant in 3/7 studies. High triglycerides were acknowledged as an essential predictor of amputation in two studies by Zubair et al. and Pemayun et al., while in one study, Li et al. found that the levels of triglycerides correlate negatively with the prediction of amputation [15-17]. CRP levels were measured in three studies only of which all three identified high levels of CRP (above 5 mg/L) as a predictive marker of amputation. Data regarding CRP in other studies were not found. In contrast, the same studies also measured the levels of erythrocyte sedimentation rate (ESR) which was found significant in two. Finally, the level of creatinine was used to assess the renal function and diagnose nephropathy as high creatinine corresponds to the presence of renal insufficiency in the studied population. Three studies recognized higher levels of creatinine in the amputation group in comparison to the non-amputation group (Figure 5).

Ulcer Characteristics

The features of the ulcers in all the patients were evaluated in order to determine the predictive significance of each and every one of them. Ulcer size was measured in diameter and was identified as significant in predicting amputation in two studies. The bigger the ulcer size, the higher the risk of amputation (mean size of more than 3 cm, p=0.001). The ulcer site was assessed in one study only which revealed that the patients who developed an ulcer on the hind foot had a higher risk of amputation with an odds ratio of (OR: 6.158, p=0.04, 95% CI: 1.808-20.974). The Wagner grade was used as a universal tool to assess the depth of the ulcer. Four studies identified Wagner grade of >3 as a predictive sign of amputation (Figure 5). Most incidences of amputation were in patients with a grade 4 ulcer. Furthermore, an ulcer complicated by osteomyelitis was a significant predictor of lower-extremity amputations (LEA) in three studies.

Co-morbidities

The included studies measured the interactions of different co-morbidities in patients with diabetic foot ulcers, and their impact on the risk of amputation. Peripheral arterial disease (PAD) was found significant in five studies making it one of the most predictive signs of amputation. Neuropathy and hypertension were significant in four studies. However, retinopathy was assessed in all seven studies and recorded poor predictive performance in all of them (Figure 5).

Discussion

Foot ulcers are a dreaded complication of diabetes as it conveys huge social and health restrictions. This systematic review inspects the performance of several risk factors in predicting amputations in diabetic foot patients, aiming to create a predictive model that contributes to the decision-making process in clinical practice. Following detailed evaluation of seven articles, the odds ratios of 28 variables were identified, of which nine were acknowledged as the strongest predictors of amputations: elevated WCC, low Hb, low albumin, high CRP and ESR, PAD, neuropathy, osteomyelitis, and Wagner grade >3. Evidence regarding other risk factors was insufficient to derive a definite association.

Most of the studies included in the review displayed an absence of any valid association between demographic factors and amputation. However, the results of some other studies not included in this review contradicted this finding, as factors like age and male gender were proven to be predictive of amputation. This finding could be attributed to the fact that they are both associated with a higher incidence of PAD [10]. In the laboratory results category, six factors appeared to be effective in predicting amputations. High WCC, ESR, and CRP as markers of infection and inflammation are strong indicators of amputation; high levels appeared to be associated with treatment failure in diabetic foot ulcers [18]. Moreover, factors like low albumin and low Hb indicate poor nutritional status and delayed wound healing therefore aggravated risk of amputation [19]. Further information regarding high HbA1c and FBG was inadequate, this is supported by a study conducted in Turkey which also found no evidence of high HbA1c as a predictor [20]. In contrast, the results of another study conducted in Malaysia revealed a great potential of high HbA1c in predicting amputations [21]. High levels of triglyceride and cholesterol were significant in three studies only, and it was attributed to the strong influence of these factors on causing cardiovascular complications like PAD. Furthermore, the levels of creatinine did not disclose any statistical significance in this review, this is explained by the use of creatinine to assess kidney function and diagnose nephropathy, which also had no predictive validity among the group of co-morbidities. These findings were consistent with other studies too [22]. PAD and neuropathy were the most effective predictors of amputation; similar results were reported in other studies [10,23-24]. Many factors were considered as predictors of amputation due to their contribution to causing PAD rather than their direct influence on the risk of amputation. Finally, nearly all of the included studies emphasized on the credibility of Wagner grade and osteomyelitis (equivalent to Wagner grade 3) in predicting amputation. The incidence of amputation was highest in Wagner grade 3 (37.6%) and Wagner grade 4 (43.92%). Wagner grade 5 had a lower incidence rate in amputations, partly due to the fact that patients must have underwent an amputation before reaching that grade. Furthermore, ulcers complicated by osteomyelitis (also equivalent to Wagner’s grade 3) had an increased risk of LEA (OR=4.04). Wagner grade provides information regarding the extent of tissue damage thus anticipates the outcome of the ulcer. This was furthermore supported by many other articles (not included in this systemic review) that were screened during the production of this review [25].

The shortcomings of this review included missing data; other factors such as the cause, location, site and size of the ulcer, alcohol, BMI, and foot deformity were not included in the analysis because the data were insufficient. Furthermore, there wasn’t enough information to calculate the sensitivity, specificity, and likelihood ratio of the predictors and diagnostic tests. Heterogeneity was evident as the studied populations differed in their size and characteristics. Finally, the review did not take psychological and behavioral factors into consideration in terms of their influence on the patient’s progression.

## Conclusions

This review aimed to establish the major predictors of lower extremity amputation in patients with diabetic foot ulcers. The evidence provided regarding certain demographics such as age and gender, as well as other glucose-related tests such as the HbA1c and fasting plasma glucose (FPG), was inconsistent and insufficient to be considered as strong predictors of LEA, according to this review. The strongest predictors found were as follows: peripheral arterial disease (PAD), diabetic neuropathy, a high Wagner’s grade, and osteomyelitis. Other useful predictors include: elevated white cell count (WCC), c-reactive protein (CRP), erythrocyte sedimentation rate (ESR), and decreased levels of hemoglobin (Hb) and albumin.
